# Up-to-date MALDI-TOF MS-based identification of the *Corynebacterium diphtheriae* species complex for improved diagnostics

**DOI:** 10.1128/jcm.01324-25

**Published:** 2026-05-22

**Authors:** Jörg Rau, Anja Berger, Alexandra Dangel, Martin Dyk, Tobias Eisenberg, Ekkehard Hiller, Christiane Hoffmann, Peter Kutzer, An Martel, Andreas Sing, Reinhard Sting

**Affiliations:** 1 Chemisches und Veterinäruntersuchungsamt Stuttgart (CVUAS) 160506, Fellbach, Germany; 2 German National Consiliary Laboratory for Diphtheria (NCLD), EU Reference Laboratory for Public Health on Diphtheria and Pertussis (EURL-PH-DIPE), WHO Collaborating Centre for Diphtheria, Bavarian Health and Food Safety Authority (LGL), Oberschleißheim, Germany; 3 NGS-Coreunit, Public Health Microbiology, Bavarian Health and Food Safety Authority (LGL), Oberschleißheim, Germany; 4 Hessian State Laboratory (LHL), Giessen, Germany; 5 Justus Liebig University Giessen, Institute of Hygiene and Infectious Diseases of Animals (IHIT) 9175, Giessen, Germany; 6 Landeslabor Berlin-Brandenburg (LLBB), Frankfurt (Oder), Germany; 7 Wildlife Health Ghent, Faculty of Veterinary Medicine, Ghent University 26656, Merelbeke, Belgium; 8 DVG-Konsiliarlabor für Corynebacterium pseudotuberculosis, Fellbach, Germany; University of California, Davis, Davis, California, USA

**Keywords:** bacterial identification, MALDI-TOF MS, MALDI-UP, validation, *Corynebacterium belfantii*, *Corynebacterium diphtheriae*, *Corynebacterium pseudotuberculosis*, *Corynebacterium ramonii*, *Corynebacterium rouxii*, *Corynebacterium silvaticum*, *Corynebacterium ulcerans *

## Abstract

The *Corynebacterium* (*C*.) *diphtheriae* species complex (*Cd*SC) comprises the closely related pathogenic species *C. diphtheriae*, *C. belfantii, C. rouxii, C. ulcerans, C. silvaticum, C. ramonii,* and *C. pseudotuberculosis* according to the taxonomic revisions after 2018. Due to their close relationship, reliable species identification within the *Cd*SC is also challenging using MALDI-TOF mass spectrometry (MS) for fast and reliable identification of bacteria. This is relevant for human and veterinary medicine. However, the Bruker MALDI-Biotyper (MBT), a commonly used MALDI-TOF MS system, does not reflect the current *Cd*SC taxonomy, which includes *C. belfantii*, *C. ramonii*, *C. rouxii*, and *C. silvaticum* as additional *Corynebacterium* species in the *Cd*SC. Therefore, the objective of the present study is to expand the commercial Bruker database version K (MBT_K_) in order to achieve reliable identification for all currently known *Corynebacterium* species within the *Cd*SC. Furthermore, the database expansions were verified with a systematic formal validation procedure in accordance with the German guideline for species identification by MALDI-TOF MS. For this, 328 spectra made from 321 strains including all seven valid species of the *Cd*SC were identified using the MBT_K_ database and a customer-supplemented modified MBT_K_ database. This custom database version included 33 reference spectra of the *Cd*SC covering all member species. The results of the formal validations for both the commercial and the extended database, combined partly with a single signal analysis, provide a substantiated basis for the identification of bacteria of the *Cd*SC. A key element for a fast up-to-date identification has been the exchange of user-generated sets of main spectrum profiles (MSPs) on the open access MALDI User Platform (MALDI-UP) catalog.

**IMPORTANCE:**

Bacteria belonging to the *Corynebacterium* (*C*.) *diphtheriae* complex are relevant pathogens for humans and animals with zoonotic potential. Identification of the novel species of this complex described since 2018, that is, *C. belfantii*, *C. rouxii*, *C. ramonii*, and *C. silvaticum*, is possible using the widely approved MALDI-TOF mass spectrometry. This is important for targeted therapies, preventive measures, and epidemiological studies. Precise species identification has been achieved by using a supplemented Bruker MALDI reference database. Comprehensive validation of database entries meets the requirements for accurate pathogen identification in accordance with recognized guidelines. This approach to community-driven database expansion and validation is certainly a model for other bacterial species of interest to react to current taxonomic revisions in a short time frame.

## INTRODUCTION

Matrix-assisted laser desorption ionization time of flight mass spectrometry (MALDI-TOF MS) is a widely used technique for the identification of microbial species in routine medical, veterinary, and food microbiology (1–3). This method is based on the comparison of mass spectra obtained from isolates in question with the reference spectra stored in a database. Although commercial databases are well organized with regard to the field of medical microbiology, this is less the case in the area of food and veterinary microbiology. This applies in particular to the zoonotic pathogens of the *Corynebacterium* (*C*.) *diphtheriae* species complex (*Cd*SC), which includes the corynebacterial groups *C. diphtheriae sensu lato* (s.l.) (*C. diphtheriae sensu stricto* (s.s.), *C. belfantii, C. rouxii*), *C. ulcerans* s.l. (*C. ulcerans* s.s., *C. silvaticum*, *C. ramonii*), and the species *C. pseudotuberculosis,* which have recently undergone a revision of the species taxonomy. *Cd*SC pathogens cause serious diseases in humans and animals, the most prominent being diphtheria, which is, therefore, subject to a strict disease notification obligation in the EU and, thus requires reliable diagnosis (4–6).

*C. diphtheriae* (7) is the eponym of the species of the group *C. diphtheriae* s.l. and the causative agent of classical diphtheria of the upper respiratory tract in humans, a potentially life-threatening, highly transmissible disease (8, 9). However, due to vaccination, classic diphtheria only occurs regionally in Europe (4, 6, 10). Taxonomically, *C. diphtheriae* has been subdivided into the two subspecies *diphtheriae* and *lausannense* (11) and the biovar Belfanti, which has recently been elevated to species rank, *C. belfantii* (12). A further closely related novel *Corynebacterium* species is *C. rouxii* (13), which was formerly assigned to *C. belfantii*. This species has so far been isolated in the context of skin infections in dogs, cats, and humans (8, 14, 15). To date, nothing is known about its zoonotic potential, so the separation of this species is also particularly relevant for epidemiological investigations.

*C. ulceran*s (16) has already been identified as a pathogen in humans and in numerous different mammalian taxa including exotic, game, pet, and livestock animals (17–24). In addition to wound infections, *C. ulcerans* also causes diphtheria-like diseases of the upper respiratory tract in humans and has surpassed the incidence of *C. diphtheriae* infections in European industrialized nations for several years (4). *C. ulcerans* infections in humans are mainly acquired through contact with animals, especially cats and dogs, and, thus, represent the most important zoonotic pathogenic agent of the *Cd*SC (18, 25–27). Isolates from wild boars previously classified as *C. ulcerans* have recently been assigned to the new species *C. silvaticum* (28). This novel species has been identified as the causative agent of caseous abscesses in wild boars, Iberian pigs, and in one reported case in a roe deer (29–32). Recently, its zoonotic potential has been proven by a few cases of human infections via contact with wild boars (33).

*C. ramonii* is a further novel zoonotic species of the group of *C. ulcerans* s.l., which affects humans, dogs, and cats (34–36).

*C. pseudotuberculosis* (37) is the causative agent of caseous lymphadenitis (CLA), a chronic infection mainly in sheep, goats, and camelids characterized by the formation of abscesses in lymph nodes and internal organs (38–42). The pathogen also causes ulcerative lymphangitis, the so called pigeon fever in horses (43). Isolates originating from water buffaloes are the only isolates known to produce diphtheria toxin and are associated with cases of edematous skin disease (OSD) (44). In general, pseudotuberculosis due to *C. pseudotuberculosis* is a rare zoonosis, which is transmitted via skin wounds after contact with affected animals (45, 46).

The correct identification of these closely related pathogenic corynebacteria according to the current taxonomy poses a challenge to diagnostics, especially the maintenance of an up-to-date and comprehensive MALDI-TOF MS database. Due to these obstacles, commercial databases usually exhibit a temporal discrepancy in this regard. In the case of species that are relevant to human and animal health, their timely inclusion is of paramount importance. Implementations of user generated solutions are able to close this diagnostic gap. In this context, it is important to emphasize the utmost relevance of quality assurance. The validation concepts that have been developed in accordance with national guidelines have already demonstrated their efficacy in regard to MALDI-TOF MS and Fourier-transform infrared spectroscopy (47–49).

This study aims to expand the database according to the recent taxonomy and intends to perform the formal validation procedure for two databases, the commercial Bruker MALDI Biotyper database version K (MBT_K_) and an adjusted MBT_K_ database, which was extended with reference spectral entries listed in the MALDI-UP catalog (MBT_K_*+MUP database). The aim is to achieve reliable identifications of the closely related and recently described taxa of the species of the *Cd*SC and their unequivocal differentiation from non-target bacteria. This procedure might also serve as a model for other scenarios of closely related bacterial species.

## MATERIALS AND METHODS

### Isolates

The type strains *C. belfantii* DSM 105776^T^, *C. diphtheriae* DSM 44123^T^, *C. pseudotuberculosis* DSM 20689^T^, *C. ramonii* CIP 112226^T^, *C. rouxii* DSM 110354^T^, and *C. ulcerans* DSM 46325^T^ were obtained from public strain collections. The type strain of *C. silvaticum* (CVUAS 4292^T^ = DSM 109167^T^) was available from the species description and previous studies (28, 29). In addition, the following isolates from publicly available strain collections were included: CIP 102462, CCM 1750, CCM 2823 (assigned as *C. ulcerans*), DSM 43988, ATCC 13812 = DSM 43989 (*C. diphtheriae*), and DSM 7177 (*C. pseudotuberculosis*). In addition to the 13 *Cd*SC strains from public strain collections, further 308 *Cd*SC field isolates were used for the generation of reference spectra and/or for validation purposes (Table S1). From these field isolates, except for one *C. diphtheriae sensu stricto* (s.s.) isolate from a horse, all utilized diagnostic isolates of *C. diphtheriae* s.s. (*n* = 26), and *C. belfantii* (*n* = 6) originated from humans and that had been sent to the German National Consiliary Laboratory for Diphtheria (NCLD; LGL Bavaria). Field isolates of *C. ulcerans* s.s. were obtained from humans (*n* = 15) and from 10 different animal species (*n* = 89). The seven *C. rouxii* isolates were obtained from canids and a hedgehog (14). *C. silvaticum* isolates (*n* = 85) were cultured from diseased wild boars and one roe deer (50). Two recent *C. silvaticum* isolates were of human origin (33). Except for the type strain of *C. ramonii,* no further assigned isolate of this species was available at the time of the start of this study. For *C. pseudotuberculosis,* 77 field isolates were included in the study. Three of these were from humans, while 74 isolates were from different ungulates (Table S1).

The human isolates included in this study had been confirmed by the NCLD by MALDI-TOF MS-based species identification (Bruker microflex with MBT_K_ database), a *tox* gene PCR, followed by an optimized modified ELEK test (51) and a recently published lateral flow diphtheria toxin (DT) immune assay (52), demonstrating DT expression if the *tox* gene PCR result was positive (53).

Phospholipase D activity (PLD) was demonstrated by the reversed CAMP phenomenon (inhibition of the outer hemolysis zone of an orthogonal growing *Staphylococcus aureus* isolate and synergistic enhancement of the hemolysis with orthogonal growing *Rhodococcus hoagii*) (cf. 50, 54).

In order to confirm the species classification of potential *C. ramonii* isolates, whole-genome DNA sequencing (WGS) was carried out, followed by an average nucleotide identity analysis using PyANI software (v. 0.2.12, https://pyani.readthedocs.io/en/latest/), an open-source python-based tool for calculating Average Nucleotide Identity (ANI). WGS was performed on a NovaSeq 6000 (S4 Reagent Kit v1.5) with 2 × 150 bp paired-end reads subsequent to Nextera XT library preparation (both Illumina, San Diego, USA), using 1 ng of genomic DNA. Library preparation and sequencing were carried out by CeGaT GmbH (Tübingen, Germany). The sequencing data sets were assembled with the AQUAMIS pipeline (55). The genomes were compared to a selection of publicly available reference genomes in the NCBI database (https://www.ncbi.nlm.nih.gov/), downloaded on 04.09.2024. The complete list of sequence data sets used can be found in the appendix (Table S2). These data sets included, for example, the *C. pseudotuberculosis* reference genome of strain MEX29 (NZ_CP016826.1) as well as *C. belfantii* FRC0043 (GCF_900205605), *C. diphtheriae* ISS3319 (NZ_CP025209.1), *C. rouxii* FRC0190 (NZ_LR738855), *C. ulcerans* 809 (NC_017317), *C. ramonii* FRC0011 (GCF_000767685.1), and *C. silvaticum* PO100/5 (NZ_CP021417). ANIm (MUMmer algorithm) and ANIb (BLASTN+ algorithm) analyses were performed with the help of PyANI as previously described by Hiller et al. (42) (Fig. S1). An ANI match with more than 95% identity to a reference isolate was used to determine the species (56).

For testing the exclusivity of the MALDI-TOF MS method, 1,264 isolates outside the *Cd*SC were used, representing 844 species of 218 genera and including 127 bacterial isolates from other members of the phylum Actinomycetota (Actinobacteria) (57). All individual tests and metadata for the isolates used are depicted in Table S1.

### Cultivation and sample preparation

Corynebacteria and other microorganisms belonging to different taxonomic groups were cultivated in accordance with their specific requirements. The individual growth conditions of all bacteria utilized for single spectra acquisitions are accessible through the MALDI-UP (MUP) catalog (58). A selection of these data is presented in Table S1. The ethanol-formate extraction protocol was used for generating MALDI-TOF mass reference spectra as recommended by the manufacturer. In addition, the direct transfer and extended direct transfer protocols were applied to record the set of individual spectra used for validation. These latter simplified protocols correspond to those used in the routine workflow in many laboratories (Table S1) (48, 59, 60). In addition, spectra listed in MUP were included, which were created using a special sample preparation procedure for highly pathogenic bacteria (61).

### MALDI-TOF MS Databases

The two databases, MBT_K_ and MBT_K_*+MUP, were used, and the results were compared.

#### Commercial MBT_K_ database

The commercial database version K for the Bruker MALDI-Biotyper Compass system (MBT_K_) was released in 2022 as a “research use only” (RUO) application (Bruker Daltonik, Bremen, Germany). This database is, therefore, not intended for *in vitro* diagnostic (IVD). The MBT_K-_ database includes 11,897 reference entries, deposited as so called main spectra or main spectra projections (MSP) by the manufacturer, which were derived from 4,320 bacterial species (database product information [62]). In the MBT_K_ database, only three species of the *Cd*SC have been included up to the year 2018: *C. diphtheriae*, *C. ulcerans,* and *C. pseudotuberculosis*. For these entries, no subspecies information has been given by the manufacturer. The names of *Cd*SC-MSPs from the MBT_K_ library entries are listed in Table 1.

**TABLE 1:** MALDI-TOF MS reference spectra of *Corynebacterium diphtheriae* species complex bacteria (*Cd*SC), available in the MALDI Biotyper database version K (MBT_K_), and additional reference spectra listed on the MALDI User Platform (MUP) used in this study^*a*^^,^^*b*^

Species	Isolate/strain	Reference entry name (MSP)	Database source	Database	
				MBT_K_	MBT_K_*+MUP
*C. belfantii*	DSM 105776^T^	*Corynebacterium belfantii* DSM 105776 CVUAS	MUP 1781		x
	KL0171	*Corynebacterium belfantii* KL0171 CVUAS	MUP 1745		x
	KL0236	*Corynebacterium belfantii* KL0236 CVUAS	MUP 1746		x
*C. diphtheriae*	DSM 44123^T^	*Corynebacterium diphtheriae* DSM 44123^T^ DSM_2	MBT_K_	x	#
		*Corynebacterium diphtheriae diphtheriae* DSM 44123 CVUAS	MUP 6620		x
	CCUG 15935	*Corynebacterium diphtheriae* CCUG 15935 CCUG	MBT_K_	x	#
	CCUG 55537	*Corynebacterium diphtheriae* CCUG 55537 CCUG	MBT_K_	x	#
	DSM 43988	*Corynebacterium diphtheriae* DSM 43988 DSM	MBT_K_	x	#
	1G12072319_3e	*Corynebacterium diphtheriae* 1G12072319_3e MVD	MBT_K_	x	#
	44_R9	*Corynebacterium diphtheriae* 44_R9 ISB	MBT_K_	x	#
	50_R5	*Corynebacterium diphtheriae* 50_R5 ISB	MBT_K_	x	#
	R1_36_29	*Corynebacterium diphtheriae* R1_36_29 ISB	MBT_K_	x	#
	KL0163	*Corynebacterium diphtheriae gravis* KL0163 CVUAS	MUP 1747		x
	KL0167	*Corynebacterium diphtheriae gravis* KL0167 CVUAS	MUP 1748		x
	KL0187	*Corynebacterium diphtheriae gravis* KL0187 CVUAS	MUP 1749		x
	KL0232	*Corynebacterium diphtheriae gravis* KL0232 CVUAS	MUP 1750		
	KL0237	*Corynebacterium diphtheriae gravis* KL0237 CVUAS	MUP 1751		x
	KL0240	*Corynebacterium diphtheriae gravis* KL0240 CVUAS	MUP 1752		x
	KL0173	*Corynebacterium diphtheriae mitis* KL0173 CVUAS	MUP 1753		x
	KL0179	*Corynebacterium diphtheriae mitis* KL0179 CVUAS	MUP 1754		x
	KL0235	*Corynebacterium diphtheriae mitis* KL0235 CVUAS	MUP 1755		x
*C. pseudotuberculosis* ‍	DSM 20689^T^	*Corynebacterium pseudotuberculosis* DSM 20689T DSM	MBT_K_	x	x
		*Corynebacterium pseudotuberculosis* Ovis gv ovis DSM 20689 CVUAS	MUP 7393		x
	59_D6_coll	*Corynebacterium pseudotuberculosis* 59_D6_coll ISB	MBT_K_	x	x
	62_D6_coll	*Corynebacterium pseudotuberculosis* 62_D6_coll ISB	MBT_K_	x	x
	64_D6_coll	*Corynebacterium pseudotuberculosis* 64_D6_coll ISB	MBT_K_	x	x
	GD7	*Corynebacterium pseudotuberculosis* GD7 GDD	MBT_K_	x	x
	GD8	*Corynebacterium pseudotuberculosis* GD8 GDD	MBT_K_	x	x
	X x 102968 C	*Corynebacterium pseudotuberculosis* x_x_102968_C IBS	MBT_K_	x	x
	992	*Corynebacterium pseudotuberculosis* Equi NTTB 992 CVUAS	MUP 935		x
	CVUAS 32689	*Corynebacterium pseudotuberculosis* Ovis gv-camelid CVUAS 32689 CVUAS	MUP 2398		x
	CVUAS 5583,2	*Corynebacterium pseudotuberculosis* bv Ovis gv-camelid CVUAS 5583.2 CVUAS	MUP 958		x
*C. ramonii*	CIP 112226^T^	*Corynebacterium ramonii* CIP 112226^T^ CVUAS	MUP 6614		x
	CVUAS 34267	*Corynebacterium ramonii* CVUAS 34,267 CVUAS	MUP 7345		x
	C104	*Corynebacterium ramonii* C104 CVUAS	MUP 7346		x
*C. rouxii*	DSM 110354^T^	*Corynebacterium rouxii* DSM 110354 CVUAS	MUP 1782		x
	191012535	*Corynebacterium rouxii* 191012535 CVUAS	MUP 1935		x
		*Corynebacterium rouxii* 191012535_LHL-GI	MUP 1744		x
	211002017-1	*Corynebacterium rouxii* 211002017-1_LHL-GI	MUP 3235		x
	211002017-2	*Corynebacterium rouxii* 211002017-2_LHL-GI	MUP 3236		x
	C138	*Corynebacterium rouxii* C138 CVUAS	MUP 813		
	CVUAS 3559,2	*Corynebacterium rouxii* CVUAS 3559,2 CVUAS	MUP 1014		x
	KL1306	*Corynebacterium rouxii* KL 1306 CVUAS	MUP 1783		
	KL1663	*Corynebacterium rouxii* KL 1663 CVUAS	MUP 1901		
*C. silvaticum*	CVUAS 4292^T^ = DSM 109166^T^	*Corynebacterium silvaticum* CVUAS 4292 CVUAS	MUP 0083		x
	CVUAS 6455	*Corynebacterium silvaticum* CVUAS 6455 CVUAS	MUP 0922		x
*C. ulcerans*	DSM 46325^T^	*Corynebacterium ulcerans* DSM 46325^T^ DSM	MBT_K_	x	#
		*Corynebacterium ulcerans* DSM 46325 CVUAS	MUP 2212		x
	66_D6_coll	*Corynebacterium ulcerans* 66_D6_coll ISB	MBT_K_	x	#
	67_D6_coll	*Corynebacterium ulcerans* 67_D6_coll ISB	MBT_K_	x	#
	71_D6_coll	*Corynebacterium ulcerans* 71_D6_coll ISB	MBT_K_	x	#
	MCW 10006	*Corynebacterium ulcerans* MCW_10006 MCW	MBT_K_	x	#
	VA22435_07	*Corynebacterium ulcerans* VA22435_07 ERL	MBT_K_	x	#
	131011719	*Corynebacterium ulcerans* NT 131011719 CVUAS	MUP 1015		x
	141001548	*Corynebacterium ulcerans* NT 141001548 CVUAS	MUP 1021		x
	07UVF148	*Corynebacterium ulcerans* 07UVF148 LLBB	MUP 0966		x
	CVUAS 10306	*Corynebacterium ulcerans* TTB CVUAS 10306 CVUAS	MUP 0945		x
	S-477-6-21	*Corynebacterium ulcerans* S-477-6-21 CVUAS	MUP 2339		x

^*a*^ MBT_K_*: Commercial database modified by extraction of entries of unclear assignment with regard to the new species of the CdSC. The extracted commercial reference spectra were marked with (#) in the last row. MUP: Custom made database entries selected from the MALDI-UP catalogue. The MUP catalogue number is given for user made entries. T: type strain; bv: biovar; gv: genomovar.

^*b*^ “x” indicates the individual reference entries in the database.

#### Updated commercial MBT_K_*+MUP database

**Fig 1:**
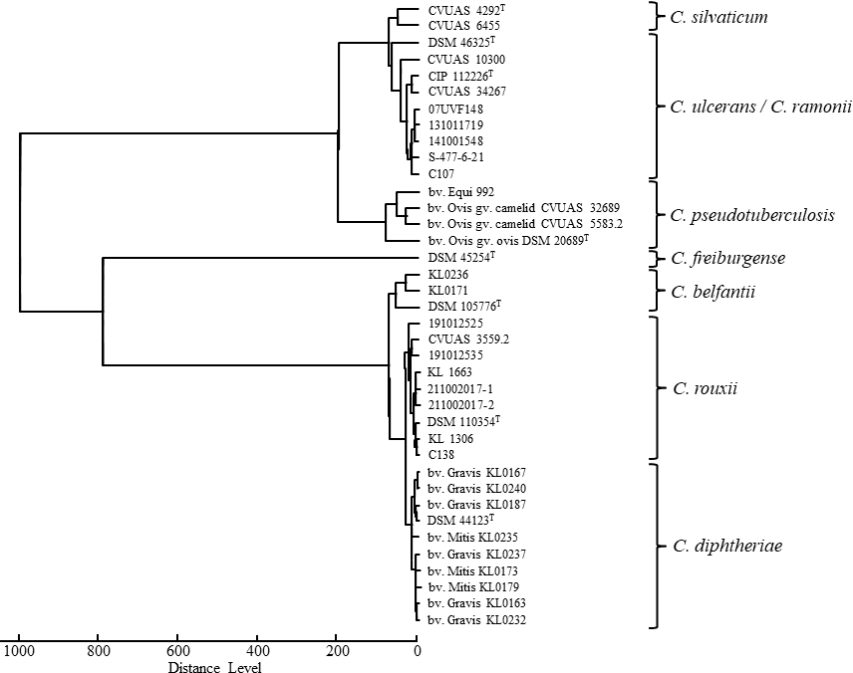
Dendrogram of selected MALDI-TOF reference mass-spectra from all current species of the *Corynebacterium diphtheriae* species complex, created by MALDI-User Platform users, depicting the spectral relationships of the species. *C. freiburgense* DSM 45254^T^ was used as an outgroup. The Cluster tool of the Biotyper Compass software was used with the default setting, using distance measures “correlation” and “average linkage”; bv: biovar; gv: genomovar.

In order to be able to identify the new species of the *Cd*SC, a revision of the MBT_K_ database was required. For this purpose, 33 customs made MSPs for all current seven *Cd*SC species were created by three laboratories in order to replace the previous MSPs of *C. diphtheriae* (s.l.) and *C. ulcerans* (s.l.) in the commercial reference database. These user-made entries were integrated into the greater MUP database using the project section of the Biotyper Compass Explorer software module (vers. 4.1.100; Bruker) (Table 1). Four further MSPs were used in the dendrogram analysis, made with the cluster tool in the Biotyper Compass Explorer software (Fig. 1). Reference spectra measurements and calculations of MSPs followed the manufacturer’s manual and commentary (60). These procedures have been recently described in more detail (48).

#### Analyses of MBT_K_ MSPs in the MBT_K_*+MUP database

As the taxonomic species which have been delineated since 2018 have not yet been included in the commercial MBT_K_ database, the MSPs of the MBT_K_ were analyzed only by the MUP database. The results were evaluated using the first two hits obtained, analogous to the evaluation of the individual validation spectra set.

### Validation spectra

For validation purposes, a total of 1,609 single MALDI-TOF mass spectra obtained from 1,585 isolates were selected from the spectra listed in the MUP catalog (version 05.03.2025, https://maldi-up.ua-bw.de) [cf. 58]. These individual spectra are raw spectra, as they occur in routine diagnostics. The metadata for all single spectra (cultivation conditions, sample preparation methods, device system, and the creator) are listed in Table S1. Of these spectra set, 457 spectra from 448 isolates belonged to the phylum Actinomycetota, including 328 spectra (321 isolates) from *Cd*SC members. The spectra used originated from pathogenic bacterial species belonging to the phylum Actinomycetota, for example, *Actinomyces* (*n* = 5 species), *Arcanobacterium* (*n* = 11), *Schaalia* (*n* = 5), or other *Corynebacterium* spp. (*n* = 37). Further information on every individual spectrum and the custom reference spectra is provided in MUP. This catalog includes metadata of the isolates, like cultivation conditions, and the data basis of species assignment of the isolate (see reference 58; Table S1). Hence, spectra from 17 laboratories were used for the complete set of spectra obtained from MUP.

### Validation procedure

The validation procedure has recently been described (48) and follows the guidelines created by the working group MALDI-TOF, pursuant to §64 of the German Food and Feed Code (47, 63). These guidelines, which are used for MALDI-TOF MS species identification in the food sector, have been adopted unchanged for the differentiation of *Cd*SC bacteria. In order to examine the effect of custom-made database additions on the identification results, two databases were compared using the same validation spectra set. The first was the commercial MBT_K_ database, and the second was a modified MBT_K_, with an exchange of the 14 commercial *Cd*SC reference entries from *C. diphtheriae* (s.l.) and *C. ulcerans* (s.l.) with 33 custom-made reference MSPs of the *Cd*SC obtained from MALDI-UP (Table 1). This version of the custom database includes 1,486 user-made entries of a broad number of bacterial species (Table 1).

Identification results were scored according to the manufacturer’s instructions (Bruker). The first result in the list has to yield a score value of at least 2.0, and the second result has to show no contradictory species that exceed a score value of >2.0. This straightforward rule was applied to the same extensive spectra set (*n* = 1,609) for both database combinations. The detailed results are presented as reports in the Tables S3a through i for the commercial MBT_K_ database and Tables S4a through f for the modified and extended database MBT_K_*+MUP.

### Manual analysis of spectra of *C. ulcerans/ C. ramonii*

**Fig 2:**
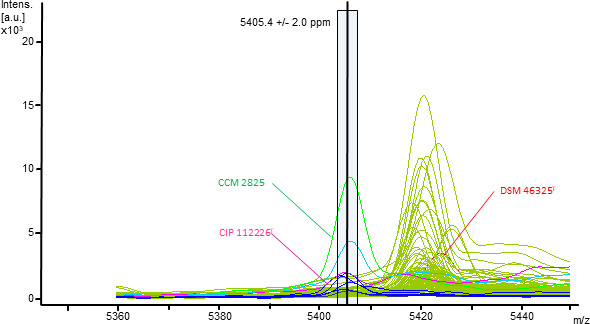
Section of the MALDI-TOF mass spectra, ranging from 5,350 to 5,450 *m/z*, displaying the signal characteristic of *Corynebacterium* (*C*.) *ramonii* at 5,405.4 *m/z*, as outlined by Crestani et al. (34). Purple: *C. ramonii* CIP 112226^T^, blue: spectra of *C. ramonii* isolates C86, C104, C139, C167, CVUAS 34267, all from hedgehogs, and pale blue and light green: KL3299A and CCM 2825 from humans, red: *C. ulcerans* DSM 46325^T^, green: overlay of single spectra of 103 *C. ulcerans* s.s. isolates (for metadata of the isolates, see Table S1). Baseline correction and smoothing of spectra were performed using settings consistent with the MSP preparation procedure. The bar shows the range of ±2 *m/z* around the specific signal. ^T^: type strain.

Crestani and colleagues observed a specific MALDI-TOF mass spectrum signal at 5,405.4 *m/z* distinguishing the newly described species *C. ramonii* from *C. ulcerans* s.s. (34). Figure 2 presents overlays of mass spectra from all *C. ulcerans* s.s. and *C. ramonii* isolates of this study, confirming the *m/z* (5,405.4 ± 2.0) window, in which the specific signal of *C. ramonii* is recognized. Therefore, raw spectra were loaded into the FlexAnalysis program (version 3.4, Bruker) for manual analysis. Baseline correction and smoothing were performed using settings consistent with the MSP preparation procedure (MBT standard method, Bruker). Peak maxima of interest were manually assigned (Fig. 2).

## RESULTS AND DISCUSSION

Numerous bacteria are currently facing significant revisions in taxonomy, promoted by a strong increase in data from WGS. Therefore, it is essential to align the widespread and accepted key tool MALDI-TOF MS for rapid and precise routine identification of bacteria to current revisions in order to ensure reliable and up-to-date species level identifications (1–3, 64). However, it must be taken into account that the precision of this tool depends on prompt database updates by the implementation of supplementary or revised reference spectra (48). Another important point is formal validation, which should be done according to accepted guidelines (47). This is particularly the case for microorganisms of medical and veterinary relevance, such as members of the closely related *Cd*SC, whose species differentiation is challenging (12, 13, 28, 34).

### The commercial MBT_K_ database

The commercial MALDI Biotyper MBT_K_ database contains reference spectra only for three representatives of the *Cd*SC (*n* = 21), including entries for the type strains of *C. diphtheriae, C. pseudotuberculosis*, and *C. ulcerans* (Table 1). However, the species described since 2018 have not yet been included in this reference data set (Bruker MBT_K_). Therefore, we created and exchanged 33 custom made reference entries to expand the commercial MBT_K_ database with the aim of improving the species identification accuracy, particularly with regard to *C. belfantii, C. ramonii, C. rouxii,* and *C. silvaticum* (Table 1; Fig. 1).

Thus, using the commercial MBT_K_ among the validation spectra set, spectra of the current taxonomic species *C. diphtheriae* s.s. (*n* = 31/31), *C. belfantii* (*n* = 7/7), and *C. rouxii* (*n* = 7/8) yielded consistent score values greater than 2.0 and were consequently supposedly assigned to “*C. diphtheriae.”* One *C. rouxii* isolate could not be identified. Considering the current taxonomy, *C. belfantii* and *C. rouxii* were not correctly identified as separate species by the MBT_K_ (Table 2).

**TABLE 2:** Species identifications of *Corynebacterium diphtheriae* species complex bacteria samples by MALDI-TOF MS using the commercial Bruker database MBT_K_^*c*^^,^^*d*^

Species/parameter of interest	No. of samples	Score mean	Score STD	No. of samples identified	Identification rate (%)	No. of true positive	No. of false negative	True positive rate (%) of identified samples	False negative rate (%) of identified samples	No. of. samples of the control group	No. of samples identified	Identification rate (%)	No. of true negative	No. of false positive	True negative rate (%) of identified samples	False positive rate (%) of identified samples	Criteria of the BVL guideline fulfilled
*C. diphtheriae* s.l.																	
*C. belfantii*	7	*2.19*	*0.09*	7	*100*	0	7^*a*^	*0*	*100^a^*	1,602	803	*50.1*	803	0	*100*	*0*	*No*
*C. diphtheriae* s.s.	31	*2.41*	*0.11*	31	*100*	31	0	*100*	*0*	1,578	786	*49.8*	772	14^*a*^	*98.2*	*1.8^a^*	*No*
*C. rouxii*	8	*2.12*	*0.11*	7	*87.5*	0	7^*a*^	*0*	*100^a^*	1,601	803	*50.2*	803	0	*100*	*0*	*No*
*C. diphtheriae* s.s.*, C. belfantii, C. rouxii* interpreted as *C. diphtheriae* s.l.	46	*2.33*	*0.16*	45	*97.8*	45	0	*100*	*0*	1,563	772	*49.4*	772	0	*100*	*0*	*Yes^a^*
*C. pseudotuberculosis*	80	*2.36*	*0.17*	80	*100*	80	0	*100*	*0*	1,529	723	*47.3*	723	0	*100*	*0*	*Yes*
*C. ulcerans* s.l.																	
*C. silvaticum*	88	*2.10*	*0.10*	78	*88.6*	0	78^*b*^	*0*	*78^b^*	1,521	803	*52.8*	803	0	*100*	*0*	*No*
*C. ramonii*	9	*2.37*	*0.09*	9	*100*	0	9	*0*	*100^b^*	1,600	803	*50.2*	803	0	*100*	*0*	*No*
*C. ulcerans* s.s.	105	*2.37*	*0.10*	105	*100*	105	0	*200*	*0*	1,504	785	*52.2*	698	87^*a*^	*88.9*	*11.1^b^*	*No*
*C. ulcerans*. s.*, C. ramonii, C. silvaticum* interpreted as *C. ulcerans* s.l.	202	*2.25*	*0.17*	202	*100*	202	0	*100*	*0*	1,485	698	*46.7*	698	0	*100*	*0*	*Yes^b^*

^*a*^ *C. belfantii *and *C. rouxii *are not represented in the database used.

^*b*^ *C. ramonii *and *C. silvaticum* are not represented in the database used. s.l.: sensu lato; s.s.: sensu stricto, No.: number; Score STD: standard deviation of the score.

^*c*^ True/False: the species was correctly/not correctly identified. All samples within the control group did not belong to the parameter of interest. Individual results for every sample are given in Tables S3a through i.

^*d*^ Italics indicate calulated values, like %, mean STD, against the counted numbers (roman).

This is also the case for the recently described species *C. ramonii* which cannot be identified by the current commercial MBT_K_ database (34). The same applies to *C. ulcerans* and *C. silvaticum* so that all validation spectra of *C. ulcerans* s.s. (*n* = 105), *C. silvaticum* (*n* = 88), and *C. ramonii* (*n* = 9) were identified as *C. ulcerans* s.l. by the commercial MBT_K_ database. In contrast, all confirmed *C. pseudotuberculosis* isolates (*n* = 80) were correctly identified using the commercial MBT_K_ with a mean score value of >2.0 (Table 2).

In summary, the commercial MBT_K_ only allows allocation to the three “classical” species *C. diphtheriae* s.l., *C. ulcerans* s.l., and *C. pseudotuberculosis*. Nevertheless, no misidentifications with non*-Cd*SC bacteria of the validation data set and no conflicting results were observed.

However, the manufacturer provides the “matching hint” with the commercial MBT_K_ database “*Species pseudotuberculosis/ulcerans of the genus Corynebacterium have very similar patterns: Therefore, distinguishing this species is difficult”* for *C. pseudotuberculosis* and *C. ulcerans* (62). Based on the results of the validation study presented here, the previous matching hint for the MBT_K_ may be replaced more appropriately by an announcement indicating the detection of *C. diphtheriae* s.l., *C. ulcerans* s.l., and *C. pseudotuberculosis* (Table 3).

**TABLE 3:** Matching hints of *Corynebacterium diphtheriae s*pecies complex samples by MALDI-TOF MS for identifications results with the commercial Bruker database MBT_K_^*a*^

Species identified by MBT_K_	Proposed matching hint to avoid misidentification
*C. diphtheriae*	This result should be interpreted as *C. diphtheriae* s.l., comprising *C. diphtheriae* s.s., *C. belfantii*, and *C. rouxii*
*C. pseudotuberculosis*	No matching hint
*C. ulcerans*	This result should be interpreted as *C. ulcerans* s.l., comprising *C. ulcerans* s.s., *C. silvaticum*, and *C. ramonii*

^*a*^ Utilising solely the commercial MBT_K_ database version, the following matching hints for the identification were proposed. Because of the lack of certain species and respective specific MSPs in the database used, a differentiation within the mentioned sensu lato groups is not applicable.

Corynebacteria not belonging to the *Cd*SC and other species of the phylum Actinomycetota as well as more distantly related bacteria included in the validation set which comprises more than 1,500 individual spectra were clearly separated from the *Cd*SC species using the commercial MBT_K_ database (Table 2).

### Identifications of MSPs from the MBT_K_ database using the MUP database

Due to the supposed uncertain species identifications of the members of the *Cd*SC according to the taxonomy after 2018, MSPs for the three classical species *C. diphtheriae*, *C. ulcerans*, and *C. pseudotuberculosis* were extracted from the MBT_K_ and identified using the MUP database. However, the identification results obtained in this way were not always consistent (Table 4). Thus, among the species attributable to *C. diphtheriae* s.l., seven of eight MSPs showed a matching result for *C. diphtheriae* s.s., consistent in the first and second hit. In contrast, the MSP “*Corynebacterium diphtheriae* 44_R9 ISB” was assigned to *C. belfantii,* a former biovar of *C. diphtheriae* and yielded scores of 2.4. Similarly, analysis of the six MBT_K_-MSPs of *C. ulcerans* s.l. resulted in several hits for the type strain of *C. ramonii* with a score of ≥2.2 as the first and/or the second hit result (Table 4).

**TABLE 4:** Database cross-check using the main spectra projections (MSPs) of the *Corynebacterium diphtheriae* species complex (*Cd*SC) from the commercial Bruker database MBT_K_ as samples, identified by using only the *Cd*SC-MSPs from the MALDI-UP catalog in order to scrutinize the recent taxonomy (cf. Table 1); bv: biovar; gv: genomovar^*a*^

Reference entry name (MSP) from MBT_K_	Result with *Cd*SC-MSP from MALDI-UP database 1. hit (score); 2. hit (score)
*Corynebacterium diphtheriae* DSM 44123T DSM_2	*C. diphtheriae gravis* KL0240 CVUAS (2.68); *C. diphtheriae gravis* KL0167 CVUAS (2.67)
*Corynebacterium diphtheriae* CCUG 15935 CCUG	*C. diphtheriae gravis* KL0163 CVUAS (2.52); *C. diphtheriae mitis* KL0235 CVUAS (2.50)
*Corynebacterium diphtheriae* CCUG 55537 CCUG	*C. diphtheriae mitis* KL0252 CVUAS (2.60); *C. diphtheriae diphtheriae* DSM 44,123 CVUAS (2.59)
*Corynebacterium diphtheriae* DSM 43988 DSM	*C. diphtheriae diphtheriae* DSM 44123 CVUAS (2.67); *C. diphtheriae gravis* KL0163 CVUAS (2.62)
*Corynebacterium diphtheriae* 1G12072319_3e MVD	*C. diphtheriae gravis* KL0167 CVUAS (2.63); *C. diphtheriae gravis* KL0237 CVUAS (2.61)
*Corynebacterium diphtheriae* 44_R9 ISB	***C. belfantii* DSM 105776 CVUAS (2.41);** ***C. belfantii* KL0171 CVUAS (2.36)**
*Corynebacterium diphtheriae* 50_R5 ISB	*C. diphtheriae gravis* KL0163 CVUAS (2.07); *C. diphtheriae mitis* KL0235 CVUAS (2.07)
*Corynebacterium diphtheriae* R1_36_29 ISB	*C. diphtheriae diphtheriae* DSM 44123 CVUAS (2.56); *C. diphtheriae mitis* KL0252 CVUAS (2.52)
*Corynebacterium pseudotuberculosis* DSM 20689T DSM	*C. pseudotuberculosis* bv Ovis DSM 20689 CVUAS (2.48); *C. pseudotuberculosis* bv Equi NTTB 992 CVUAS (2.230)
*Corynebacterium pseudotuberculosis* 59_D6_coll ISB	*C. pseudotuberculosis* bv Ovis gv-camelid CVUAS 5583.2 CVUAS (2.53); *C. pseudotuberculosis* bv Ovis gv-camelid CVUAS 32689 CVUAS (2.47)
*Corynebacterium pseudotuberculosis* 62_D6_coll ISB	*C. pseudotuberculosis* bv Ovis gv Camelid CVUAS 5583.2 CVUAS (2.36); *C. pseudotuberculosis* bv Ovis gv Camelid CVUAS 32689 CVUAS (2.31)
*Corynebacterium pseudotuberculosis* 64_D6_coll ISB	*C. pseudotuberculosis* bv Ovis gv Camelid CVUAS 5583.2 CVUAS (2.37); *C. pseudotuberculosis* bv Ovis DSM 20689 CVUAS (2.29)
*Corynebacterium pseudotuberculosis* GD7 GDD	*C. pseudotuberculosis* bv Ovis gv Camelid CVUAS 5583.2 CVUAS (2.54); *C. pseudotuberculosis* bv Ovis gv Camelid CVUAS 32689 CVUAS (2.47)
*Corynebacterium pseudotuberculosis* GD8 GDD	*C. pseudotuberculosis* bv Ovis DSM 20689 CVUAS (2.51); *C. pseudotuberculosis* bv Ovis gv Camelid CVUAS 5583.2 CVUAS (2.48)
*Corynebacterium pseudotuberculosis* x_x_102968_C IBS	*C. pseudotuberculosis* bv *Ovis* DSM 20689 CVUAS (2.24); *C. pseudotuberculosis* bv Ovis gv Camelid VUAS 5583.2 CVUAS (2.22)
*Corynebacterium ulcerans* DSM 46325T DSM	***C. ramonii* CVUAS 34267 CVUAS (2.45);** *C. ulcerans* NT 131011719 CVUAS (2.32)
*Corynebacterium ulcerans* 66_D6_coll ISB	*C. ulcerans* NT 141001548 CVUAS (2.12); *C*. *ulcerans* 07UVF148 LLBB (2.12)
*Corynebacterium ulcerans* 67_D6_coll ISB	***C. ramonii* CVUAS 34267 CVUAS (2.27);** *C. ulcerans* NT 131011719 CVUAS (2.21)
*Corynebacterium ulcerans* 71_D6_coll ISB	*C. ulcerans* DSM 46325 CVUAS (2.25); *C. ulcerans* NT 141001548 CVUAS (2.21)
*Corynebacterium ulcerans* MCW_10006 MCW	***C. ramonii* CVUAS 34267 CVUAS (2.64);** ***C. ramonii* CIP 112226 CVUAS (2.61)**
*Corynebacterium ulcerans* VA22435_07 ERL	***C. ramonii* CVUAS 34267 CVUAS (2.41);** ***C. ramonii* CIP 112226 CVUAS (2.38)**

^*a*^ In bold: divergent results for the MBT_K_ reference spectra.

This cross-check investigation revealed that representatives of the new *Cd*SC species may be masked among the earlier reference entries in the commercial MBT_K_ Bruker database. Thus, MSPs of *C. ulcerans* (s.l.) and *C. diphtheriae* (s.l.) were excluded from the commercial MBT_K_ database, in order to eliminate any potential inaccuracies in classification (Table 4). This is mentioned as the “modified” MBT_K_*.

The single spectra identified as *C. ulcerans* s.l., using the MBT_K_ database, were examined for the single signal at *m/z* 5,405.4 described by Crestani et al. (34) as a unique signal marker for *C. ramonii* (34). In addition to the type strain, seven isolates exhibited a positive signal for this marker (see Fig. 2). The aforementioned conspicuous isolates were subsequently analyzed in detail by whole-genome sequencing (WGS), following the approach described before (42). This analysis assigned the wildlife isolates C86, C104, C139, C167, CVUAS 34267 originating from hedgehogs, and the human isolates KL3299A and CCM 2823 to *C. ramonii*. Once the isolates mentioned above had been reassigned, the two databases were subsequently validated. In contrast, no conflicting results were identified for the well delimited species *C. pseudotuberculosis*.

Consequently, an optimized database (MBT_K_*+MUP database) enriched by novel MSP expansions and a selective subtraction of suboptimal MSPs from the commercial database (MBT_K_) is used.

### The updated commercial database MBT_K_*+MUP

The custom set of reference spectra from the MUP database (Fig. 1), and the application of the described specific *m/z* values, for *C. ulcerans/C. ramonii*, refined and improved the identification results for all *Cd*SC species. Thus, using the MBT_K_*+MUP database, all spectra from *C. diphtheriae* s.s. isolates were correctly assigned to the corresponding species (31 out of 31). In comparison to the use of the MBT_K_ database, the mean score for this set of spectra could be increased from 2.19 to 2.45. Isolates reliably assigned to *C. belfantii* (type strain and several isolates previously identified as *C. diphtheriae* bv. Belfanti at the NCLD) were correctly identified at the species level with the new database, using the first two hit approach. Furthermore, all *C. rouxii* spectra (*n* = 8) were also correctly identified with the MBT_K_*+MUP database (Table 5). Within *C. diphtheriae* s.s., biovar information was available only for some isolates, but not for the subspecies. This aspect was, therefore, not investigated.

**TABLE 5:** Results of species identification of *Corynebacterium diphtheriae* species complex samples by MALDI-TOF MS using the modified database (MBT_K_*+MUP)^*a*^^,^^*b*^

Species/parameter of interest	No. of samples	Score mean	Score STD	No. of samples identified	Identification rate (%)	No. of true positive	No. of false negative	True positive rate (%) of identified samples	False negative rate (%) of identified samples	Samples of the control group	No. of samples identified	Identification rate (%)	No. of true negative	No. of false positive	True negative rate (%) of identified samples	False positive rate (%) of identified samples	Criteria of the BVL guideline fulfilled
*C. belfantii*	7	*2.49*	*0.16*	7	*100*	7	0	*100*	*0*	1,602	1,225	76.5	1,225	0	*100*	*0*	*No*
*C. diphtheriae* s.s.	31	*2.45*	*0.17*	30	*100*	30	0	*100*	*0*	1,578	1,221	77.4	1,221	0	*100*	*0*	*Yes*
*C. rouxii*	8	*2.62*	*0.15*	8	*100*	8	0	*100*	*0*	1,601	1,243	77.6	1,243	0	*100*	*0*	*No*
*C. pseudotuberculosis*	80	*2.46*	*0.14*	80	*100*	80	0	*100*	*0*	1,529	1,171	76.6	1,171	0	*100*	*0*	*Yes*
*C. silvaticum*	88	*2.52*	*0.12*	88	*100*	88	0	*100*	*0*	1,521	1,163	76.5	1,163	0	*100*	*0*	*Yes*
*C. ulcerans–C. ramonii*	114	*2.47*	*0.10*	114	*100*	114	0	*100*	*0*	1,495	1,179	78.9	1,179	0	*100*	*0*	*Yes*
Species decision by single peak analysis (*m/z* 5,405.4)																	
*C. ramonii*	9			9	*100*	9	0	*100*	*0*	105		100	105	0			*No*
*C. ulcerans* s.s.	105			105	*100*	105	0	*100*	*0*	9		100	9	0			*Yes*

^*a*^ True/False: the species was correctly/not correctly identified. All samples within the control group did not belong to the parameter of interest. Individual results for every sample are given in Tables S4a through f. s.l.: sensu lato; s.s.: sensu stricto, No.: number; Score STD: standard deviation of the score.

^*b*^ Italics indicate calulated values, like %, mean STD, against the counted numbers (roman).

Similarly, all spectra of *C. ulcerans* s.s. isolated from a broad collection of host species were correctly assigned with the MBT_K_*+MUP database. The 88 isolates of *C. silvaticum*, corresponding to the former “wild-boar cluster” of *C. ulcerans* (50), were also correctly identified and no discrepancies to *C. ulcerans* or *C. ramonii* were observed with the modified database. The two species, *C. ulcerans* s.s. and *C. ramonii*, were combined in the formal validation step (Table S4f; Table 5). Presently, only the subsequent evaluation of the species-specific *m/z* value of 5,405.4 for *C. ramonii* described by Crestani et al. (34) allows a separation from *C. ulcerans* s.s. All isolates’ spectra showing the respective signal were confirmed as belonging to *C. ramonii* by WGS analysis (Fig. S1). Remarkably, also strain CCM 2823 (= CIP 54.69) was provisionally assigned to *C. ramonii*. Due to the small number of available isolates of *C. ramonii* (*n* = 9), a formal validation, which requires at least 20 individual spectra for the validation of a targeted identification in the sense of the guideline is pending (47).

Regarding *C. pseudotuberculosis*, the use of the modified and extended MBT_K_*+MUP database shows no divergence of the number of correct assignments and thus no apparent advantage compared to the commercial MBT_K_ database. However, using the MBT_K_*+MUP database, a moderately higher mean score value was obtained for the validation spectra set used (2.36 vs 2.46).

No divergent identifications were observed for other species of the genus *Corynebacterium* (*n* = 37), other Actinomycetota species (*n* = 57), or even more distantly related microorganisms (*n* = 750) included in the validation data set (Table 2).

Overall, *C. diphtheriae, C. pseudotuberculosis, C. silvaticum,* and *C. ulcerans* can be reported as validated parameters in our accredited laboratories for official veterinary diagnostics. This achievement is based on the fulfillment of all requirements of the extended reference spectra collection (MBT_K_^*^ +MUP) and the validation procedure according to specified formal criteria of the BVL technical guidelines (47). For *C. belfantii, C. ramonii,* and *C. rouxii*, the prospects of differentiation are promising although the number of available spectra for isolates of these species is still too small for a complete formal validation.

For all the seven species of the *Cd*SC, we propose identification hints using the commercial MBT_K_ database to guide the user and to avoid erroneous interpretation of the results (Table 3).

Using the MBT_K_*+MUP database, the identification of species not belonging to the *Cd*SC is enhanced significantly (Tables 2 and 5). In this case, the identification rate of the control group increases from 47.3% for all isolate spectra (*n* = 1,529) using the commercial MBT_K_ database to 76.6% using the custom MBT_K_*+MUP database (e.g., Table S3e vs Table S4e). Therefore, we conclude that regular database updates are necessary to ensure a comprehensive, efficient, and consistent up-to-date identification. Subsequently, the custom MBT_K_*+MUP database was in use for first specific studies (10).

As previously demonstrated, the user-driven exchange of custom entries accessible on MUP serves as an effective approach for a vast expansion of in-house identifications, complementing the periodic updates of the commercial database (48). The availability of open access catalogs, like MUP, facilitates a global inter-laboratory exchange of spectra. Moreover, the option of downloading spectra collections, such as the RKI database, enables users to freely access and provide data for a broad scientific community (65, 66). Finally, successful validation can be performed based on a broad collection of well-documented MALDI-TOF MS single spectra to reduce the workload for each single laboratory (47, 48).

### Conclusions and prospects

MALDI-TOF MS has become an indispensable tool for a fast and reliable identification of bacteria at the species level in many diagnostic laboratories. However, a sophisticated and up-to-date database is necessary for the exact identification of closely related bacterial species according to current taxonomy, as here demonstrated for the valid species of the *Cd*SC. Thus, database users should be aware of possible performance flaws with ongoing taxonomic changes. Consequently, the identification of bacteria according to the current taxonomy is the basis of treatment of human and animal infections, risk assessment, and the implementation of preventive measures such as vaccination programs.

Using *Cd*SC as an example, here we show for the first time taking into account current guideline, how to keep up with the latest taxonomic assignments of bacteria by modifying and extending a current commercial database with own and exchanged MSPs. In this regard, the globally free accessible MALDI-UP catalog for user created reference and validation spectra was utilized and the employed validation process used in this study fulfills the required number of isolates in accordance with the applied guidelines (47). This approach to the identification of very closely related bacterial species with recent taxonomic changes using MALDI-TOF MS can certainly be used as a blueprint for other sophisticated bacterial identifications in order to react to complex species identifications of interest in a short time frame. Using appropriate methods, AI could support the manual evaluation of spectra and data in the future, to reduce the formal validation effort shown here.

### Highlights

- Using MALDI-TOF MS, all species of the *C. diphtheriae* species complex are accurately identified according to the current taxonomy in order to adequately address the requirements of epidemiology and zoonosis control
- Validation was performed using spectra from different institutions and an approved standard protocol
- The expansion and the exchange of user created reference spectra via the MALDI User Platform are proved to be a key tool for precise identification of bacteria

## SUPPLEMENTAL MATERIAL

Table S1 jcm.01324-25-s0001.xlsx MALDI - Validation Spectra.

Table S2 jcm.01324-25-s0002.xlsx *Corynebacterium* Reference Genomes.

Tables S3a-S3i jcm.01324-25-s0003.xlsx Reports of Validation_targeted-ID_MBTk (11897).

Tables S4a-S4f jcm.01324-25-s0004.xlsx Reports of Validation_targeted-ID_MBTk_MUP (13370).

Figure S1 jcm.01324-25-s0005.pdf Heatmap showing the average nucleotide identity (ANI) analysis of 184 *Corynebacterium* isolates.

Supplemental legends jcm.01324-25-s0006.docx Legends for Tables S1, S2, S3a-i, S4a-f, and Figure S1.

ASM does not own the copyrights to Supplemental Material that may be linked to, or accessed through, an article. The authors have granted ASM a non-exclusive, world-wide license to publish the Supplemental Material files. Please contact the corresponding author directly for reuse.
