# Supplementary material for: Up-to-date MALDI-TOF MS-based identification of the Corynebacterium diphtheriae species complex for improved diagnostics
Source: J Clin Microbiol. 2026 May 22;64(6):e01324-25. doi: 10.1128/jcm.01324-25 (PMC13251421; doi:10.1128/jcm.01324-25)
Supplement: Supplemental legends — Legends for Tables S1, S2, S3a-i, S4a-f, and Figure S1. [file jcm.01324-25-s0006.docx]

Up-to-date MALDI-TOF MS based identification of the *Corynebacterium diphtheriae* species complex for improved diagnostics

**Jörg Rau^1^, Anja Berger^3^, Alexandra Dangel^8^, Martin Dyk^1^, Tobias Eisenberg^4^, Ekkehard Hiller^1^, Christiane Hoffmann^5^, Peter Kutzer^6^, An Martel^7^, Andreas Sing^3^, Reinhard Sting^1,2^**

# Supplemental Material - Legends

#### Table S1

Isolates and spectra used.

Data extract from MALDI User Platform (MUP; version 10.02.2025), with metadata of all isolates used in this study and with technical metadata for the individual MALDI-TOF mass spectra used.

Reference:
CVUAS. 2015. Chemisches und Veterinäruntersuchungsamt Stuttgart: MALDI-TOF MS user platform MALDI-UP. Retrieved from [https://www.maldi-up.ua-bw.de](https://www.maldi-up.ua-bw.de/), Accessed 10th Feb 2025.

#### Table S2

List of reference genomes for ANI downloaded from NCBI.

#### Figure S1

Heatmap showing the average nucleotide identity (ANI) analysis of 184 *Corynebacterium* isolates

#### Table S3a-S3i

#### Reports of validations for Corynebacterium (C.) identification of species from the C. diphtheriae species complex with MALDI-TOF MS using the Bruker MALDI Biotyper database MBT_K_.

Reference:
BVL. 2022. Federal Office of Consumer Protection and Food Safety: Guidelines for validating species identifications using matrix-assisted laser desorption/ionisation time-of-flight mass spectrometry (MALDI-TOF-MS) in a single laboratory or in laboratory networks, pp. 1-32 <https://www.bvl.bund.de/SharedDocs/Downloads/07_Untersuchungen/Guidelines_for_validating_species_identifications_using_MALDI-TOF-MS.pdf;jsessionid=385A5C363D8718FF3D44B0A04694F886.2_cid372?__blob=publicationFile&v=4>.

#### Table S3a

Validation of *Corynebacterium* (*C.*) *belfantii* identification with MALDI-TOF MS using the Bruker MALDI Biotyper database MBT_K_ – Report.

Quantitative evaluation of the MALDI Biotyper, database version K (MBT_K_; Bruker Daltonics) classification performance: results for all single spectra of *C. belfantii* strains and for the non-parameter (i.e. non-*C. belfantii*) dataset, following the principles of the Guidelines for Validating Species Identifications for Targeted Parameters (BVL, 2022).

#### Table S3b

Validation of *Corynebacterium* (*C.*) *diphtheriae* s.s. identification using the Bruker MALDI Biotyper database MBT_K_ – Report.

Quantitative evaluation of the MALDI Biotyper, database version K (MBT_K_; Bruker Daltonics) classification performance: results for all single spectra of *C. diphtheriae* s.s. strains and for the non-parameter (i.e. non-*C. diphtheriae* s.s.) dataset, following the principles of the Guidelines for Validating Species Identifications for Targeted Parameters (BVL, 2022).

#### Table S3c

Validation for *Corynebacterium* (*C.*) *rouxii* identification using the MALDI Bruker Biotyper database MBT_K_ – Report.

Quantitative evaluation of the MALDI Biotyper, database version K (MBT_K_; Bruker Daltonics) classification performance: results for all single spectra of *C. rouxii* strains and for the non-parameter (i.e. non-*C. rouxii*.) dataset, following the principles of the Guidelines for Validating Species Identifications for targeted parameters (BVL, 2022).

#### Table S3d

Validation for *Corynebacterium* (*C.*) *diphtheriae* s.l. identification using the Bruker MALDI Biotyper database MBT_K_ – Report.

Quantitative evaluation of the MALDI Biotyper, database version K (MBT_K_; Bruker Daltonics) classification performance: results for all single spectra of *C. diphtheriae* s.l. strains and for the non-parameter (i.e. non-*C. diphtheriae* s.l.) dataset, following the principles of the Guidelines for Validating Species Identifications for targeted parameters (BVL, 2022).

#### Table S3e

Validation for *Corynebacterium* (*C.*) *pseudotuberculosis* identification using the Bruker MALDI Biotyper database MBT_K_ – Report.

Quantitative evaluation of the MALDI Biotyper, database version K (MBT_K_; Bruker Daltonics) classification performance: results for all single spectra of *C. pseudotuberculosis* strains and for the non-parameter (i.e. non-*C. pseudotuberculosis*) dataset, following the principles of the Guidelines for Validating Species Identifications for targeted parameters (BVL, 2022).

#### Table S3f

Validation for *Corynebacterium* (*C.*) *silvaticum* identification with MALDI-TOF MS using the Bruker MALDI Biotyper database MBT_K_ – Report.

Quantitative evaluation of the MALDI Biotyper, database version K (MBT_K_; Bruker Daltonics) classification performance: results for all single spectra of *C. silvaticum* strains and for the non-parameter (i.e. non-*C. silvaticum*) dataset, following the principles of the Guidelines for Validating Species Identifications for targeted parameters (BVL, 2022).

#### Table S3g

Validation for *Corynebacterium* (*C.*) *ramonii* identification with MALDI-TOF MS using the Bruker MALDI Biotyper database MBT_K_ - Report.

Quantitative evaluation of the MALDI Biotyper, database version K (MBT_K_; Bruker Daltonics) classification performance: results for all single spectra of *C. ramonii* strains and for the non-parameter (i.e. non-*C. ramonii*) dataset, following the principles of the Guidelines for Validating Species Identifications for targeted parameters (BVL, 2022).

#### Table S3h

Validation for *Corynebacterium* (*C.*) *ulcerans* identification with MALDI-TOF MS using the Bruker MALDI Biotyper database MBT_K_ - Report.

Quantitative evaluation of the MALDI Biotyper, database version K (MBT_K_; Bruker Daltonics) classification performance: results for all single spectra of *C. ulcerans* strains and for the non-parameter (i.e. non-*C. ulcerans*) dataset, following the principles of the Guidelines for Validating Species Identifications for targeted parameters (BVL, 2022).

#### Table S3i

Validation for *Corynebacterium* (*C.*) *ulcerans* s.l. identification using the Bruker MALDI Biotyper database MBT_K_ – Report.

Quantitative evaluation of the MALDI Biotyper, database version K (MBT_K_; Bruker Daltonics) classification performance: results for all single spectra of *C. ulcerans* s.l. strains and for the non-parameter (i.e. non-*C. ulcerans* s.l.) dataset, following the principles of the Guidelines for Validating Species Identifications for targeted parameters (BVL, 2022).

#### Table S4a-S4f

#### Reports of validations for Corynebacterium (C.) identification of species of the C. diphtheriae species complex with MALDI-TOF MS using the modified Bruker MALDI Biotyper database MBT_K_* combined with the MALDI-UP expansion.

Reference:
BVL. 2022. Federal Office of Consumer Protection and Food Safety: Guidelines for validating species identifications using matrix-assisted laser desorption/ionisation time-of-flight mass spectrometry (MALDI-TOF-MS) in a single laboratory or in laboratory networks, pp. 1-32 <https://www.bvl.bund.de/SharedDocs/Downloads/07_Untersuchungen/Guidelines_for_validating_species_identifications_using_MALDI-TOF-MS.pdf;jsessionid=385A5C363D8718FF3D44B0A04694F886.2_cid372?__blob=publicationFile&v=4>.

#### Table S4a

Validation of Co*rynebacterium* (*C.*) *belfantii* identification using the modified Bruker MALDI Biotyper database MBT_K_* combined with the MALDI-UP expansion – Report.

Quantitative evaluation of the MALDI Biotyper combined with a database expansion (MBT_K_*+MUP) classification performance: results for all single spectra from *C. belfantii* strains and for the non-parameter (i.e. non-*C. belfantii*) isolate set, following the principles of the Guidelines for Validating Species Identifications for targeted parameters (BVL, 2022).

#### Table S4b

Validation of Co*rynebacterium* (*C.*) *diphtheriae* s.s. identification using the modified Bruker MALDI Biotyper database MBT_K_* combined with the MALDI-UP expansion - Report.

Quantitative evaluation of the MALDI Biotyper combined with a database expansion (MBT_K_*+MUP) classification performance: results for all single spectra from *C. diphtheriae* s.s. strains and for the non-parameter (i.e. non-*C. diphtheriae s.s.*) isolate set, following the principles of the Guidelines for Validating Species Identifications for targeted parameters (BVL, 2022).

#### Table S4c

Validation of Co*rynebacterium* (*C.*) *rouxii* identification using the modified Bruker MALDI Biotyper database MBT_K_ *combined with the MALDI-UP expansion – Report.

Quantitative evaluation of the MALDI Biotyper combined with a database expansion (MBT_K_*+MUP) classification performance: results for all single spectra from *C. rouxii* strains and for the non-parameter (i.e. non-*C. rouxii*) isolate set, following the principles of the Guidelines for Validating Species Identifications for targeted parameters (BVL, 2022).

#### Table S4d

Validation of Co*rynebacterium* (*C.*) *pseudotuberculosis* identification using the modified Bruker MALDI Biotyper database MBT_K_* combined with the MALDI-UP expansion - Report.

Quantitative evaluation of the MALDI Biotyper combined with a database expansion (MBT_K_*+MUP) classification performance: results for all single spectra from *C. pseudotuberculosis* strains and for the non-parameter (i.e. non-*C. pseudotuberculosis*) isolate set, following the principles of the Guidelines for Validating Species Identifications for targeted parameters (BVL, 2022).

#### Table S4e

Validation of Co*rynebacterium* (*C.*) *silvaticum* identification using the modified Bruker MALDI Biotyper database MBT_K_* combined with the MALDI-UP expansion - Report.

Quantitative evaluation of the MALDI Biotyper combined with a database extension (MBT_K_*+MUP) classification performance: results for all single spectra from *C. silvaticum* strains and for the non-parameter (i.e. non-*C. silvaticum*) isolate set, following the principles of the Guidelines for Validating Species Identifications for targeted parameters (BVL, 2022).

#### Table S4f

Validation of Co*rynebacterium* (*C.*) *ulcerans-ramonii* identification using the modified Bruker MALDI Biotyper database MBT_K_* combined with the MALDI-UP expansion - Report.

Quantitative evaluation of the MALDI Biotyper combined with a database expansion (MBT_K_*+MUP) classification performance: results for all single spectra from *C. ulcerans-ramonii* strains and for the non-parameter (i.e. non-*C. ulcerans-ramonii*) isolate set, following the principles of the Guidelines for Validating Species Identifications for targeted parameters (BVL, 2022).
