# Supplementary material for: Up-to-date MALDI-TOF MS-based identification of the Corynebacterium diphtheriae species complex for improved diagnostics
Source: J Clin Microbiol. 2026 May 22;64(6):e01324-25. doi: 10.1128/jcm.01324-25 (PMC13251421; doi:10.1128/jcm.01324-25)
Supplement: Figure S1 — Heatmap showing the average nucleotide identity (ANI) analysis of 184 Corynebacterium isolates. [file jcm.01324-25-s0005.pdf]

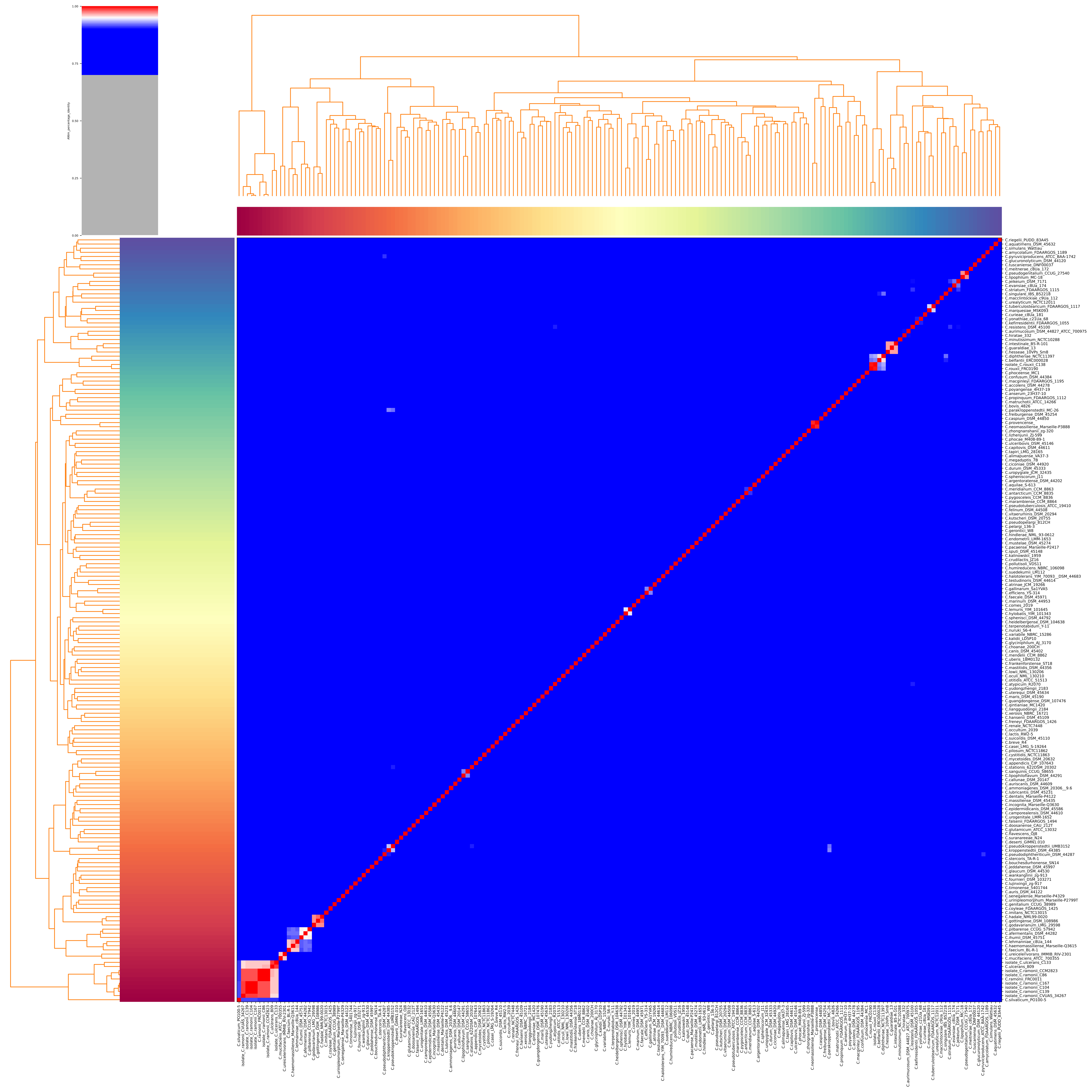

ANIm_percentage_identity
1.00
0.75
0.50
0.25
0.00
C.riegelii_PUDD_83A45
C.aquatimens_DSM_45632
C.simulans_Wattiau
C.amycolatum_FDAARGOS_1189
C.pyruviciproducens_ATCC_BAA-1742
C.glucuronolyticum_DSM_44120
C.tuscaniense_DNF00037
C.meitnerae_c8Ua_172
C.pseudogenitalium_CCUG_27540
C.lipophilum_MC-18
C.jeikeium_DSM_7171
C.evansiae_c8Ua_174
C.striatum_FDAARGOS_1115
C.singulare_IBS_B52218
C.macclintockiae_c9Ua_112
C.urealyticum_NCTC12011
C.tuberculostearicum_FDAARGOS_1117
C.marquesiae_MSK093
C.curieae_c8Ua_181
C.yonathiae_c21Ua_68
C.kefirresidentii_FDAARGOS_1055
C.aurimucosum_DSM_44827_ATCC_700975
C.resistens_DSM_45100
C.hiratae_332
C.minutissimum_NCTC10288
C.intestinale_BS-R-101
C.guaraldiae_13
C.hesseae_10VPs_Sm8
C.diphtheriae_NCTC11397
C.belfantii_ERC00028
isolate_C.rouxii_C138
C.rouxii_FRC0190
C.phoceense_MC1
C.confusum_DSM_44384
C.macginleyi_FDAARGOS_1195
C.accolens_DSM_44278
C.poyangense_4H37-19
C.anserum_23H37-10
C.propinquum_FDAARGOS_1112
C.matruchotii_ATCC_14266
C.bovis_4826
C.parakroppenstedtii_MC-26
C.freiburgense_DSM_45254
C.caspium_DSM_44850
C.provencense_
C.neomassiliense_Marseille-P3888
C.zhongnanshanii_zg-320
C.lizhenjunii_ZJ-599
C.phocae_M408-89-1
C.ulceribovis_DSM_45146
C.capitovis_DSM_44611
C.tapiri_LMG_28165
C.alimapuense_VA37-3
C.megadyptis_78
C.ciconiae_DSM_44920
C.durum_DSM_45333
C.uropygiale_JCM_32435
C.spheniscorum_J11
C.argentoratense_DSM_44202
C.aquilae_S-613
C.meridianum_CCM_8863
C.antarcticum_CCM_8835
C.pygosceleis_CCM_8836
C.marambiense_CCM_8864
C.pseudotuberculosis_ATCC_19410
C.felinum_DSM_44508
C.vitaeruminis_DSM_20294
C.kutscheri_DSM_20755
C.pseudopelargi_812CH
C.pelargi_136-3
C.gerontici_W8
C.hindlerae_NML_93-0612
C.endometrii_LMM-1653
C.mustelae_DSM_45274
C.pacaense_Marseille-P2417
C.sputi_DSM_45148
C.kalinowskii_1959
C.crudilactis_JZ16
C.pollutisoli_VDS11
C.humireducens_NBRC_106098
C.suedekumii_LM112
C.halotolerans_YIM_70093__DSM_44683
C.testudinoris_DSM_44614
C.atrinae_JCM_19266
C.gallinarum_Sa1YVA5
C.efficiens_YS-314
C.faecale_DSM_45971
C.marinum_DSM_44953
C.comes_2019
C.lemuris_YIM_101645
C.hylobatis_YIM_101343
C.sphenisci_DSM_44792
C.heidelbergense_DSM_104638
C.terpenotabidum_Y-11
C.nuruki_S6-4
C.variabile_NBRC_15286
C.kalidii_LD5P10
C.glyciniphilum_AJ_3170
C.choanae_200CH
C.canis_DSM_45402
C.mendelii_CCM_8862
C.uberis_18M0132
C.frankenforstense_ST18
C.mastitidis_DSM_44356
C.lowii_NML_130206
C.oculi_NML_130210
C.otitidis_ATCC_51513
C.atypicum_R2070
C.yudongzhengii_2183
C.uterequi_DSM_45634
C.maris_DSM_45190
C.guangdongense_DSM_107476
C.qintianiae_MC1420
C.liangguodongii_2184
C.xerosis_NBRC_16721
C.hansenii_DSM_45109
C.freneyi_FDAARGOS_1426
C.renale_NCTC7448
C.occultum_2039
C.lactis_RW2-5
C.suicordis_DSM_45110
C.breve_R4
C.casei_LMG_S-19264
C.pilosum_NCTC11862
C.cystitidis_NCTC11863
C.mycetoides_DSM_20632
C.appendicis_CIP_107643
C.stationis_622DSM_20302
C.sanguinis_CCUG_58655
C.lipophiloflavum_DSM_44291
C.callunae_DSM_20147
C.auriscanis_DSM_44609
C.ammoniagenes_DSM_20306__9.6
C.lubricantis_DSM_45231
C.dentalis_Marseille-P4122
C.massiliense_DSM_45435
C.incognita_Marseille-Q3630
C.epidermidicanis_DSM_45586
C.camporealensis_DSM_44610
C.urogenitale_LMM-1652
C.falsenii_FDAARGOS_1494
C.doosanense_CAU_212T
C.glutamicum_ATCC_13032
C.flavescens_OJ8
C.suranareeae_N24
C.deserti_GIMN1.010
C.pseudokroppenstedtii_UMB3152
C.kroppenstedtii_DSM_44385
C.pseudodiphtheriticum_DSM_44287
C.stercoris_TA-R-1
C.bouchesdurhonense_SN14
C.oedipnesis_DSM_45997
C.glaucum_DSM_44530
C.wankanglinii_zg-913
C.fournieri_DSM_103271
C.lujinxingii_zg-917
C.timonense_5401744
C.auris_DSM_44122
C.senegalense_Marseille-P4329
C.uriniipleomorphum_Marseille-P2799T
C.genitalium_CCUG_38989
C.coyleae_FDAARGOS_1425
C.imitans_NCTC13015
C.hadale_NML99-0020
C.gottingense_DSM_108986
C.godavarianum_LMG_29598
C.pilbarense_CCUG_57942
C.afermentans_DSM_44282
C.thomssenii_DSM_45751
C.lehmanniae_c8Ua_144
C.haemomassiliense_Marseille-Q3615
C.faecium_BL-R-1
C.ureicelerivorans_IMMIB_RIV-2301
C.mucifaciens_ATCC_700355
C.ulcerans_809
isolate_C.ulcerans_C133
isolate_C.ramonii_CCM2823
C.ramonii_FRC0011
isolate_C.ramonii_C86
isolate_C.ramonii_C167
isolate_C.ramonii_C104
isolate_C.ramonii_C139
isolate_C.ramonii_CVUAS_34267
C.silvaticum_PO100-5
